# Analyzing Nursing Home Complaints: From Substantiated Allegation to Deficiency Citations

**DOI:** 10.1093/geroni/igaa057.271

**Published:** 2020-12-16

**Authors:** Kallol Kumar Bhattacharyya, Lindsay Peterson, John Bowblis, Kathryn Hyer

**Affiliations:** 1 University of South Florida, Tampa, Florida, United States; 2 Miami University, Oxford, Ohio, United States

## Abstract

Complaints provide important information to consumers about nursing homes (NHs). Complaints that are substantiated often lead to an investigation and potentially a deficiency citation. The purpose of this study is to understand the relationship between substantiated complaints and deficiency citations. Because a complaint may contain multiple allegations, and the data do not identify which allegation(s) lead to a complaint’s substantiation, we identified all substantiated single allegation complaints for NHs in 2017. Our data were drawn from federally collected NH complaint and inspection records. Among the 369 substantiated single-allegation complaints, we found most were categorized as quality of care (31.7%), resident abuse (17.3%), or resident neglect (14.1%). Of the deficiency citations resulting from complaints in our sample, 27.9% were categorized as quality of care and 19.5% were in the category of resident behavior and facility practices, which includes abuse and neglect. While two-thirds (N=239) of the substantiated complaints generated from 1 to 19 deficiency citations, nearly one third had no citations. Surprisingly, 28% of substantiated abuse and neglect allegations resulted in no deficiency citations. More surprisingly, a fifth of complaints that were categorized as “immediate jeopardy” at intake did not result in any deficiency citations. We also found a number of asymmetries in the allegation categories suggesting different processes by Centers for Medicare and Medicaid Services (CMS) region. These results suggest that the compliant investigation process warrants further investigation. Other policy and practice implications, including the need for better and more uniform investigation processes and staff training, will be discussed.

